# Food Marketing Influences Children’s Attitudes, Preferences and Consumption: A Systematic Critical Review

**DOI:** 10.3390/nu11040875

**Published:** 2019-04-18

**Authors:** Rachel Smith, Bridget Kelly, Heather Yeatman, Emma Boyland

**Affiliations:** 1 Early Start, School of Health and Society, Faculty of Social Sciences, University of Wollongong, Northfields Ave, Wollongong, NSW 2522, Australia; bkelly@uow.edu.au; 2 School of Health and Society, Faculty of Social Sciences, University of Wollongong, Northfields Ave, Wollongong, NSW 2522, Australia; hyeatman@uow.edu.au; 3 Psychological Sciences, Institute of Psychology, Health and Society, University of Liverpool, Bedford Street South, Liverpool L69 7ZA, UK; e.boyland@liverpool.ac.uk

**Keywords:** systematic review, food marketing, childhood obesity, marketing techniques, vehicles of marketing

## Abstract

Exposure to the marketing of unhealthy foods and beverages is a widely acknowledged risk factor for the development of childhood obesity and noncommunicable diseases. Food marketing involves the use of numerous persuasive techniques to influence children’s food attitudes, preferences and consumption. This systematic review provides a comprehensive contemporary account of the impact of these marketing techniques on children aged 0–18 years and critically evaluates the methodologies used. Five electronic academic databases were searched using key terms for primary studies (both quantitative and qualitative) published up to September 2018; 71 eligible articles were identified. Significant detrimental effects of food marketing, including enhanced attitudes, preferences and increased consumption of marketed foods were documented for a wide range of marketing techniques, particularly those used in television/movies and product packaging. Together, these studies contribute strong evidence to support the restriction of food marketing to children. However, the review also signposted distinct gaps: Firstly, there is a lack of use of qualitative and physiological methodologies. Secondly, contemporary and sophisticated marketing techniques used in new media warrant increased research attention. Finally, more research is needed to evaluate the longer-term effects of food marketing on children’s weight.

## 1. Introduction

Globally, the prevalence of overweight and obesity has risen dramatically amongst children aged 5–19 years, from 4% in 1975 to 18% in 2016 [1]. As obesity in childhood is known to track into adulthood [2], this highlights a cohort of 41 million children with the potential to become adults with overweight and obesity with serious implications for health. Obesity, a disease in itself, is also a modifiable behavioural risk factor for long-term noncommunicable diseases (NCDs), such as cardiovascular diseases and some cancers [3,4]; thus, early intervention is critical. 

Obesity is arguably a natural response to the modern food environment [5], where the marketing and advertising of inexpensive, highly palatable, energy-dense foods and beverages is omnipresent [5,6]. The techniques used to market unhealthy foods to children are extensive, sophisticated and persuasive [7,8] and target different vehicles of promotion (e.g., television) via varying marketing techniques (e.g., product placement). Analysis of children’s environments indicates persuasive marketing has a particularly strong presence on television [9], websites [10] and games [11] and extends its promotion to supermarkets [12] and outside schools [13], resulting in minimal uncommercialised space. 

Children are particularly susceptible to persuasive messages used in marketing communications, as their cognitive development (cognition that allows one to recognise the selling and persuasive intent of marketing communications) is relatively limited [14]. For example, young children in the ‘pre-cognition’ stage of cognitive defence are unable to differentiate television advertising and television programme content [15]. Children are more likely to interpret marketing as factual [16]. Younger children are known to interpret advertising as assistive information to ensure they are up-to-date with what is available in the shops [16]. Therefore, it could be argued marketing exploits children’s cognitive limitations and so is both inherently unfair [17] and a breach of children’s rights to appropriate information as stated in Article 17 of the United Nations Convention for the Rights of the Child [18].

Acute and accumulative exposures to food marketing influence children’s thoughts and behaviours, in particular, their attitudes, preferences and consumption of unhealthy commodities [19,20,21]. These factors are key components of a cascade of effects of marketing that may lead to childhood overweight [22]. The most recent systematic assessment of the evidence on all three of these proximal outcomes, published in 2013, included literature up to 2009 [23]. Thus, there is nearly a decade of new research to be evaluated. Moreover, previous studies have noted methodological gaps in the body of evidence, such as a need for child-appropriate study measures and in-depth qualitative data to complement quantitative information [22]. Recent meta-analyses focused on consumption outcomes also noted substantial methodological heterogeneity for the quantitative studies [20,24]. 

Therefore, this review aimed to provide a contemporary account of the impact of food marketing on children’s food attitudes, preferences and consumption. It also aimed to explore the methodologies used in the identified studies to determine salient gaps in the research. The key areas of enquiry in this review informed the following research questions:

(1) What are the impacts of different food marketing techniques on children’s (0–18 years) food attitudes, preferences and consumption?
(2) What methodologies and marketing techniques have been studied to evaluate the impact of food marketing on children’s (0–18 years) food attitudes, preferences and consumption?
(3) Are there opportunities to further explore the impact of food marketing techniques on children’s (0–18 years) food attitudes, preferences and consumption?

## 2. Materials and Methods 

The systematic review protocol was registered with the PROSPERO International Prospective Register of Systematic Review [25] prior to inception (ID CRD42018107429) and is reported in accordance with the Preferred Reporting Items for Systematic Reviews and Meta-Analyses (PRISMA) [26]. The lead researcher (RS) independently searched the following five academic electronic databases during September 2018: SCOPUS (https://www.scopus.com/), PsycINFO (http://www.apa.org/pubs/databases/psycinfo/index.aspx), MEDLINE (https://www.ncbi.nlm.nih.gov/pubmed/), Business Source Complete (https://www.ebsco.com/products/research-databases/business-source-complete), and Web of Science (http://apps.webofknowledge.com). The following Boolean search strings were used: (market* OR persua* OR advert* OR commercial OR promot* OR technique OR brand*) AND (child* OR adolescen* OR “young people” OR teen OR “junior high” OR “primary school” OR “high school” OR “secondary school” OR youth OR boys OR girls OR camp OR parent*) AND (food OR drink OR beverage OR snack OR juice OR soda NOT alcohol*) AND (consum* OR attitude OR choice OR intake OR prefer*). Articles were required to adhere to the following criteria: They were peer-reviewed journal articles, published in English, and were published in the period 1970–2018. Whilst the review focused on outcomes measured in 0–18-year-olds, parents were included in the search terms to account for studies in which they may have responded on behalf of young children or completed assessments/questionnaires. A manual search of reference sections in eligible articles supplemented the formal electronic searches.

### 2.1. Study Selection

**Figure 1:**
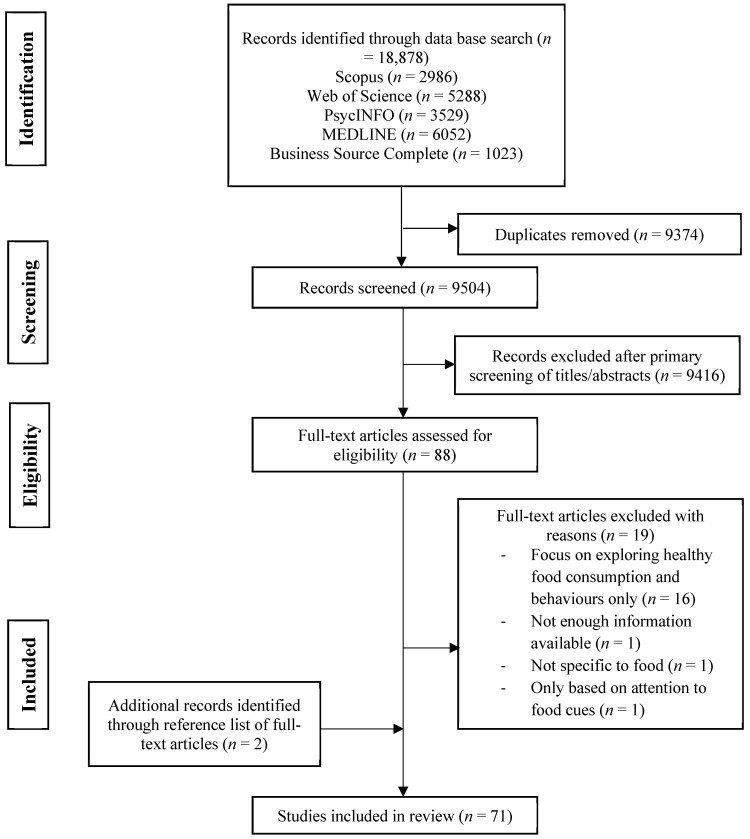
PRISMA flow chart of the systematic review literature search.

Eligibility criteria required primary research that explored the influence of one or more marketing technique(s) on one or more of children’s (0–18) attitudes, preferences or consumption of food or beverages. Criteria extended to both quantitative and qualitative primary studies to capture all methodologies. Figure 1 illustrates the systematic literature search. Potential material was exported into EndNote X8 [27], and duplicates were removed. The lead reviewer (RS) prescreened the title and abstract of the identified references for relevance. Secondary studies such as systematic reviews and content analysis articles were not included. Exclusion criteria precluded studies that: Focused on examining marketing techniques to promote good nutrition or studies focused on outcomes other than attitudes, preference or consumption such as purchasing requests or body weight. Two independent reviewers (RS and a research assistant) assessed each of the full-text articles against the inclusion and exclusion criteria. A third reviewer (BK) was consulted when there were discrepancies between the two reviewers, and a consensus was reached through discussion. 

### 2.2. Data Extraction

RS and a research assistant extracted and recorded data in a tabulated summary. Details recorded included the date, location, objectives, study design, sample size, demographics, procedure, main marketing technique evaluated, outcome measures, and primary outcomes. Due to the heterogeneity of the studies, a qualitative narrative synthesis was used to communicate the overall conclusions of the studies. RS and a research assistant both separately conducted a quality assessment on the selected articles using the NIH (National Institute of Health) tools [28] for quantitative studies, and the CASP (Critical Appraisal Skills Program) [29] for qualitative studies. Each appraisal tool assessed four domains: Study setup, participant selection, assessment and data analysis, and each study was given a rating of either good, fair or poor. 

## 3. Results

The database searches identified 18,878 records, of which 9504 remained after the removal of duplicates. Seventy-one articles met the inclusion criteria (see Figure 1). 

### 3.1. Marketing Techniques Evaluated

Six key marketing techniques and platforms for marketing were identified (broad topics included: Television/movies, packaging, digital games, endorsers, print advertising and the internet). Table 1 documents the outcomes assessed; outcomes spanned the three proximal outcomes of interest and the outcome ‘preference’ included studies which assessed preference by choice from a selection of items. More detailed data extraction tables are included in the Supplementary Tables S1–S6. 

### 3.2. Description of Studies

Study participants’ ages ranged from 2–17 years, and nearly all studies used a mixed-sex sample, with the exception of one study that was males only [44]. The majority of studies were conducted in North America (*n* = 23), followed by the UK (*n* = 14), Australia (*n* = 9), the Netherlands (*n* = 6), Canada (*n* = 4) and 12 other countries. Sixty-eight studies used a quantitative methodology and three studies used a qualitative methodology (indicated in Table 1). The quantitative studies employed either between subjects (*n* = 52) or within subjects (*n* = 16) designs. A majority of the quantitative studies measured acute (one-time) exposures to food advertising (*n* = 62), and a few investigated the impact of cumulative food marketing exposure (*n* = 6), with exposure periods ranging from 2–16 days. The three qualitative studies implemented interviews with friendship pairs [94] and one-on-one interviews [74,82].

### 3.3. Coverage and Impact of Marketing Techniques

Table 1 indicates the three most commonly studied marketing techniques involved television/movies (*n* = 36), packaging (*n* = 22) and digital games (*n* = 13). The impact of television commercials, particularly the influence of exposure on preferences and consumption, had been studied the most (*n* = 31). Common outcomes of exposure to television and movie marketing of unhealthy foods included consuming significantly more advertised and non-advertised food (relative to those who were not exposed or were shown healthier alternatives/nonfood advertisements) and increased positive attitudes toward and more frequent choices of the advertised food or unhealthier foods. For example, a television commercial study, deemed to be of high quality, embedded food or toy commercials into a television programme and provided participants with the opportunity for ad libitum snacking during the show with the inclusion of the advertised food [56]. It revealed that participants who viewed the food advertisements consumed an average of 48 kilocalories more of the advertised food than those who viewed toy advertisements (*p* < 0.01) [56]. 

A substantial number of studies on the influence of packaging were identified, particularly with a focus on preference (including food choice) (*n* = 22). Many of these studies demonstrated the persuasive nature of promotional characters and labelling used on the packaging of food products. For example, a study (given a high-quality rating in this review) asked children to rate their taste preferences and preferred snack choice for three matched food pairs, presented either with or without brand-equity characters (characters developed specifically to represent a brand) on the packaging [64]. Children were significantly more likely to prefer the taste (*p* < 0.1) and were more likely to choose the item in packaging with these characters (73% of children) compared with a matched food without the characters (*p* < 0.001) [64]. 

The impact of digital games was the third most frequently evaluated marketing technique (*n* = 13) and was explored chiefly by exposing children to advergames. Advergames provide a brand-rich video game environment [98], where the brand and/or product is a prominent feature [99]. They have become a popular platform for advertisers to connect with children online [100]. The evidence base for the influence of food marketing within advergames has a nearly equal number of studies for the measurement of all three outcomes. The studies used advergames with durations ranging from 2–12 min, commonly followed by an assessment for attitudes and the opportunity for ad libitum snacking. The marketing of unhealthy foods through advergames significantly increased children’s consumption of unhealthy food (*p* < 0.03) [84], and when children were exposed to both advergames and television commercials, advergames generated the most positive brand attitudes (*p* < 0.001) [34]. 

Some of the studies in Table 1 also investigated factors that may mediate the impact of marketing techniques such as: A protective message [91], attentional bias [92], weight status [47,75], genetics [56], parental influence [40,41,54], developmental stage and gender [38,40] and health knowledge [39]. These studies indicated a fast latency of initial fixations to food cues (*p* = 0.05), heavier weight status (*p* = 0.05 and *p* = 0.04) and FTO risk alleles (*p* = 0.02) all increased food consumption in children [47,56,75,92]. Food marketing was also more likely to influence the food preferences of boys than girls (*p* = 0.03) [38]. Some findings showed a moderating impact of and the activation of health knowledge [39] (*p* = 0.03), but overall, the influence of food marketing was not mediated by a protective message (Dutch children *p* = 0.1 and Spanish children *p* = 0.2) [91], parental influence (*p* > 0.05, *p* > 0.05 and *p* = 0.7) [40,41,54] or age (*p* = 0.3) [38]. 

### 3.4. Gaps in Marketing Techniques Explored

Fewer studies investigated the impact of endorsers (*n* = 4), print advertising (*n* = 3) or the internet (*n* = 2), and only one study measured the outcome of consumption. The sole study that explored the impact of endorsers on consumption was given a high-quality rating in this review and found the presence of a sports endorser led children to consume significantly more food than children who were not exposed to the endorsed material (*p* < 0.001) [93]. The available studies on print advertising and the internet predominantly revealed a strong influence of these techniques on children’s attitudes and preferences. For example, one study (given a fair rating in this review) investigated the impacts of magazine advertisements and found children exposed to food advertisements in the magazines were more likely to choose the advertised items when making a subsequent food choice compared to those who saw no food advertising (*p* = 0.04) [95]. Two studies explored the impact of internet advertisements and the use of social media but revealed mixed findings on the influence on children’s preferences (*p* < 0.001) [49] and food choice (*p* = 0.6) [97]. Lastly, it is worth noting that whilst there were more studies focused on the influence of digital gaming, the methodology used was limited because only exposure to advergames was measured, i.e., investigating the impact of exposure to games with food or brands embedded into the game itself (presence or absence). Thus, no research investigated other techniques used in online games played by children.

### 3.5. Design and Methodological Gaps

Many of the studies took similar approaches to the measurement of attitudes, preferences and consumption, such as administering questionnaires or scales, allowing the participants to make a real or hypothetical food choice, and measuring ad libitum intake of provided foods. Very few studies investigated these outcomes using extraneous methods, such as measuring physiological outcomes of exposure. Three studies implemented additional measures to investigate the potential mechanisms underpinning exposure effects [37,69,92]. For example, eye-tracking has been used to measure attentional bias, in which an interaction between the type of advergame and the latency of initial fixation to food cues influenced consumption of snacks [92]. Children with a faster latency of initial fixations in the energy-dense advergame had a higher total intake (189.2 kcal) than those who played the nonfood advergame (131.2 kcal) (*p* < 0.05) [92]. Functional resonance imaging has also been applied as a measure to understand food choice. It has shown increased brain activity in a reward region of the brain when children were making food choices after food marketing exposure (*p* < 0.05) [37]. 

The review identified a strong emphasis on quantitative studies, with only three qualitative studies available [74,82,94]. A study given a high rating in this review used friendship pairs and revealed that children claimed to like licensed characters on products because “all my favourite stuff is on it” [94]. This is a useful insight and could be used, for example, in complementing the experimental studies that have shown promotional characters are influential on children’s preferences [64,65,68,69,71]. 

There was a deficiency of studies that explored the long-term impact of marketing techniques on any outcome (*n* = 6). One study, given a high rating by this review, exposed children to either television commercials or a combination of television commercials plus advergames (multimedia) over a period of 6 days [58]. This study successfully detected that children did not compensate for eating more after exposure to advertising, leading to an additional daily food intake of 194kj (*p* < 0.001) [58]. It revealed all children in the multiple-media condition ate 182kJ more (*p* < 0.01) compared to children who were exposed to a single-media source [58]. 

The majority of the studies in this review investigated the influence of marketing unhealthy food products using stimuli selected by the researchers (*n* = 70). This did not guarantee the stimuli used would appeal to the participant sample. Studies often used well-known brands and marketing content commonly used to promote to children, such as that retrieved from well-known advertisements, shown during television hours popular with children. Only one study showed the participants a selection of brands and asked them to identify the brands they had an interaction or connection with in the real world, which were then used as the study stimuli [65]. Even in this case, the researchers had decided the selection of brands from which the children would choose. Similarly, few studies used brands assumed to be unfamiliar to the children (*n* = 2), demonstrated to be useful to investigate the effect of exposure in the absence of existing associations or preferences. 

## 4. Discussion

Overall, the identified studies present a strong, comprehensive body of evidence demonstrating the powerful influence of food marketing exposure. The studies also identify the influences on children’s attitudes, preferences and consumption of the vehicles of promotion and associated techniques, particularly with regard to television commercials, and the marketing techniques used in packaging of products. The review signposts the vehicles of promotion and marketing techniques that require further assessment and the importance of further research to strengthen the current body of evidence.

A lack of evidence linking food marketing to childhood obesity is an oft-cited reason, by both governments [101,102] and the food industry [103], for the limited action to restrict children’s exposure to unhealthy food marketing. This review of the body of evidence indicates otherwise. It has documented a strong link between food marketing to childhood obesity. The findings of this review support further restriction of food marketing to children as a key solution for the management of childhood obesity [21,104]. 

### 4.1. Strengths and Weaknesses of Selected Studies

The majority of the reviewed studies were assigned either good or fair ratings. The studies’ main strengths were in the exposure and assessment methods used. Their weaknesses were most visible in the participant sample used (i.e., not using random sequence generation, a less-generalisable sample, and nonreporting of sufficient statistical power in the sample size). There is a possibility of publication bias that studies which did not find any signification associations may not have been published; however, evidence shows that this was formally tested with intake studies in 2016 and no evidence was found of publication bias [20].

### 4.2. Priority Areas Identified for Future Research

This review identified areas of future research to strengthen the body of evidence. These consisted of additional methodology and additional marketing techniques which are detailed in Table 2. 

Marketing communications aim to influence children’s thoughts and behaviours via both the implicit and explicit memory; thus, some messages are consciously recognised when processed, and some are processed automatically without conscious awareness. Therefore, for researchers seeking to quantify the behavioural impact of marketing, it is crucial that the methodology used be appropriate to the type of exposure assessed and the relevant measurable outcome. For example, for overt marketing, it may be most appropriate to capture explicit articulation or attitude ratings, but with covert marketing exposure, it may be beneficial to observe physiological behavioural responses. This is especially relevant as studies noted an increasing use of marketing techniques designed to influence children’s implicit memory [105,106]. Research into these implicit and physiological responses is extremely valuable for expanding knowledge about the individual and automatic responses food marketing can prompt. Research with children, who may have more difficulty expressing themselves than adults [107,108], requires appropriate methodology and the use of a range of implicit and explicit techniques to ensure findings are not reliant on the children explicitly expressing their reaction. Such studies are vital for attaining a holistic understanding of the impact of food marketing on children. In this review, very few studies implemented a physiological measure to evaluate the implicit influence of food marketing. This is a notable gap in the available evidence.

It is also necessary, particularly when conducting research with children, to explore motivations and reasoning through means other than experimental studies, for example, with the use of qualitative studies. Qualitative and child-centred methodology can help children to feel meaningfully involved [109] and may allow researchers to tease apart what specific aspects of marketing resonate most with children. This review only identified three studies that used qualitative methodology. Future research incorporating more qualitative research will add greater insight and weight to the body of evidence. 

In all studies, researchers had deemed the exposure stimuli as potentially appealing to children. The materials were often popular global or national brands children were likely to have seen before. This highlights an opportunity for future research. Firstly, children have been described as ‘experts in their own lives’ [110] and may be the most accurate source for determining appropriate stimuli, especially for studies that seek to measure the impact of exposure so as to contribute to policy evidence at a global level. This is not to say the studies made a mistake in choosing the stimuli, but more studies should champion the use of stimuli informed by the participants, in turn using brands and products considered most relevant to their participants. Secondly, a majority of studies used brands assumed popular with and recognisable by with children. Using unfamiliar brands ensures measurement of exposure outcomes occurs in the absence of existing brand associations and preferences. Future research should also seek to use unfamiliar or mock food brands as stimuli. 

Furthermore, future research should measure the impacts of accumulative exposure, reflective of the longer-term effects of food marketing and children’s exposure to repeated promotions in real life. Two studies in this review investigated whether or not children compensate for advertising-induced snack consumption at subsequent meals [52,58]. The results identified potential links between advertising and longer-term body weight and health outcomes, evidence vital for informing global policymaking.

Additional marketing techniques for future research foci are of a contemporaneous nature, which likely explains why new media appear to be an understudied area of food marketing. Content analyses examining digital platforms have discovered a vast amount of marketing on popular children’s websites [111,112] and food brand websites [112,113]. However, this review identified very few studies explored the effect of marketing on websites or other digital platforms. The studies exploring social media and internet advertisements found these forms of marketing to children had detrimental consequences for dietary health, and this warrants further research. This is not without methodological and ethical challenges that make identifying and replicating what children are exposed to online a hurdle to overcome [114], yet the evidence generated is imperative for informing contemporary global policymaking. 

Further to a need for more research into the digital environment, there was a bias identified in the approach studies used to measure the influence of online games, as all studies implemented an advergame model. Advergames are very common [115] and are very influential on children, if not more influential than television commercials when compared on the same participants [34,58]. Therefore, understanding and recognising their influence is vital. However, children are not thought to spend much of their online time on food brand websites where advergames are housed [116]. It is believed “gaming” as an online phenomenon is on the rise [117], and the games children play contain numerous contemporary advertising techniques, such as pop-up and unlock-to-play advertisements [118]. Future research should seek to explore these techniques and establish their impacts, to ensure academic knowledge synchronises with the contemporary marketing environment. 

## 5. Conclusions

This review found a strong body of evidence that exposure to food marketing impacts children’s attitudes, preferences and consumption of unhealthy foods, with detrimental consequences to health. Current studies provide valuable insights and provide compelling evidence to support the restriction of food marketing to children. Future research to explore contemporaneous marketing techniques, using a wider range of methodologies, could further strengthen this body of evidence. 

## Figures and Tables

**Table 1:** Marketing technique or vehicle of marketing studied, outcome(s) assessed and the quality of the evidence.

Marketing Technique or Vehicle of Marketing	Outcome Assessed		
	Attitudes	Preferences (including Food Choice)	Consumption
**Television/Movies**			
Television commercials for unhealthy products	[30,31,32,33,34,35]	[31,32,34,35,36,37,38,39,40,41,42,43,44,45,46,47,48,49,50,51]	[31,35,44,45,46,47,52,53,54,55,56,57,58]
Product placement/movie tie-ins	[59,60]	[59,60,61,62]	[57,60]
**Packaging**			
Promotional characters	[63,64,65,66]	[50,64,65,67,68,69,70,71]	[71]
Branding		[72,73] [74]_qual_	[75,76]
Toys			[77,78]
Labelling/colour		[67,79,80,81] [82]_qual_	
**Digital games**			
Advergames	[33,34,83,84,85,86,87,88]	[34,84,85,87,89,90]	[58,84,87,88,91,92]
**Endorsers**			
Celebrities	[66]	[79]	[93]
Animated characters		[90]	
**Print advertising**			
Magazines	[94]_qual_ [95]	[95,96]	
**Internet**			
Social media		[49]	
Online advertisements		[97]	

This table contains both quantitative and qualitative studies. Qualitative studies are indicated by ‘qual’.

**Table 2:** Research gaps to be addressed in future studies.

Additional Marketing Techniques	Additional Methodology
Contemporary marketing techniques and vehicles of marketing: - Social media - Internet advertising - Advertising in online games (i.e., pop-up advertisements) - Other new media	Explicit and implicit techniques. These may involve: - Qualitative methods - Child-centred methods - Physiological methods
	Stimuli - Stimuli informed by participants - Unfamiliar stimuli
	Exposure duration - Accumulative exposures

## Supplementary Materials

The following are available online at https://www.mdpi.com/2072-6643/11/4/875/s1, Table S1: Digital Games, Table S2: Endorsers, Table S3: Internet, Table S4: Packaging, Table S5: Print Advertising, Table S6: Television/movies.
