# Supplementary material for: Food Marketing Influences Children’s Attitudes, Preferences and Consumption: A Systematic Critical Review
Source: Nutrients. 2019 Apr 18;11(4):875. doi: 10.3390/nu11040875 (PMC6520952; doi:10.3390/nu11040875)
Supplement: Supplementary file 1 [file nutrients-11-00875-s001.zip › Supplementary Files/Supplementary table S1-Digital Games.docx]

Digital Games

| **Author (year), country** | **Title** | **Sample size** | **Participant characteristics (sex, age)** | **Main marketing technique/vehicle used** | **Outcome measures** | **Primary outcomes/themes** | **Quality Assessment** |
| --- | --- | --- | --- | --- | --- | --- | --- |
| Folkvord et al. (2013), The Netherlands | The effect of playing advergames that promote energy-dense snacks or fruit on actual food intake among children | 270 | Mixed, 8-10 years | Advergame | BMI   Energy intake   Food choice   Hunger   Brand attitude | - Playing an advergame containing food cues increased general energy intake, regardless of the advertised brand or product type (energy-dense snacks or fruit) (p < .01), and this activity particularly increased the intake of energy-dense snack foods (p < .01)  - Children who played an advergame promoting food [energy-dense snacks (p < .01) or fruit (p < .01)] ate much more than did the children who played the non-food advergame.  - Children who played an advergame that promoted food [energy-dense snacks (p < .01) or fruit (p < .01)] also ate significantly more than did the children in the control condition.  - Sex (p < .05), hunger (p < .01), and age (p < .05) were significantly related to energy-dense calorie intake. Male children, children who reported being hungry, and younger children ate more energy-dense snacks. | Good |
| Folkvord et al. (2014), The Netherlands | Impulsivity, “advergames,” and food intake | 261 | Mixed, 7-10 years | Advergame | Impulsivity   BMI   Energy intake   Hunger | - Playing an advergame promoting energy-dense snacks contributes to increased caloric intake in children (p < .01) - The advergame promoting energy-dense snacks overruled the inhibition task to refrain from eating among impulsive children, making it more difficult for them to refrain from eating (p < .01) | Good |
| Folkvord et al. (2015), The Netherlands | The role of attentional bias in the effect of food advertising on actual food intake among children | 92 | Mixed, 7-10 years | Advergame | Energy intake   Attentional bias   Hunger | - The results showed that playing an advergame containing food cues increased total intake (p < .05)  - Children with a higher gaze duration for the food cues ate more of the advertised snacks (p < .05) - Children with a faster latency of initial fixation to the food cues ate more in total and ate more of the advertised snacks (p < .05)  - The number of fixations on the food cues did not increase actual snack intake. | Good |
| Folkvord et al. (2017), The Netherlands and Spain | Does a ‘protective’ message reduce the impact of an advergame promoting unhealthy foods to children? An experimental study in Spain and The Netherlands | 597   Netherlands = 215    Spain = 382 | Mixed, mean age: Spain = 8.9  Netherlands = 9 | Advergames | Hunger    Energy intake   BMI   Memory of a protective message | - Playing an advergame promoting energy-dense snacks increased caloric intake Dutch children (p <.001) but not Spanish children (p = 0.4)  - This was not mediated by a ‘protective’ message (Dutch children p = 0.1 and Spanish children p = 0.2)  - Only 5% of the Dutch children and 4% of the Spanish children who  played the energy-dense advergame with the ‘protective’ message remembered the text of the ‘protective’ message | Good |
| Harris et al. (2012), United States | US food company branded advergames on the internet: Children's exposure and effects on snack consumption | 152 | Mixed, 7-12 years | Advergame | Food choice   Energy intake  Media use | - Children consumed significantly more unhealthy food in unhealthy advergame condition (p < .03)  - Children consumed significantly more healthy food in the healthy advergame condition (p < .02) | Fair |
| Mallinckrodt et al. (2013), United States | The effects of playing an advergame on young children's perceptions, preferences, and requests | 295 | Mixed, 5-8 years | Advergame | Brand preference  Recognizing the commercial nature of an advergame   Understanding the intent of web promotion   Brand knowledge   Intention to request   Intention to pester  Liking of advergame | - More children (65%) in the treatment group tended to prefer Froot Loops to the other cereal options, compared with the children (35%) in the control group (p < .02).  - Seven (p < .001) and eight-year-olds (p < .001) in the treatment group tended to prefer Froot Loops cereal compared with children of those ages in the control group  - There was no support for an advergame generating more requests to get the brand in the game from children playing the game, compared with those not playing the game. | Good |
| Neyens et al. (2017), United Kingdom | Transferring game attitudes to the brand: persuasion from age 6 to 14 | 940 | Mixed, 6-14 years | Advergame | Brand attitude   Intention to pester   Brand preferences    Brand recognition Persuasion knowledge    Source recognition   Source intent   Attitudes towards the specific advertising format | - Children who played the advergame reported significantly more positive **brand attitudes** compared to children who had watched the TV ad (p <.001), and children in the no advertising exposure control group (p < .03).  - For pester intent, the impact of the advertising format was also significant (p < .05).  - Pester intent was significantly higher for the advergame than the TV ad (p < .02), but not compared to the control group (p = 0.36. | Fair |
| Norman et al. (2018), Australia | Sustained impact of energy-dense TV and online food advertising on children’s dietary intake: a within-subject, randomised, crossover, counter-balanced trial | 160 | Mixed, 7-12 years | Advergame | Brand recognition  BMI  Energy intake | - All children in the multiple-media condition ate more at a snack after exposure to food advertising compared with non-food advertising; this was not compensated for at lunch, leading to additional daily food intake of 194 kJ (p < .001).  - Exposure to multiple-media food advertising compared with a single-media source increased the effect on snack intake by a difference of 182 kJ (p < .01)  - Food advertising had an increased effect among children with heavier weight status in both media groups. | Good |
| Panic et al. (2013), Belgium | Comparing TV ads and advergames targeting children: the impact of persuasion knowledge on behavioural responses | 254 | Mixed, 7-10 years | Advergame | Attitude toward the advergame   Intention to request   Source recognition   Source intent  Persuasion knowledge | · **Children** liked the **advergame** (with cue) significantly more than the **TV** advertisement (p < .001)  - In the **TV** advertisement condition, the results show a significant negative effect of **persuasion knowledge** on purchase request (p <.03). | Fair |
| Putnam et al. (2018), United States | Character apps for children's snacks: effects of character awareness on snack selection and consumption patterns | 132 | Mixed, 4-5 years | Media characters (in apps) | Food choice   Awareness of character | - An ordered logistic regression found no significant effect of treatment conditions compared with the control group. - Within treatment conditions, awareness of the character led to selection and consumption of more healthy snacks in the healthier condition (p < .01), and of unhealthy snacks in the unhealthy condition (p < .03), but children were unaware that the character influenced their decisions. | Good |
| Redondo et al. (2012), Spain | The effectiveness of casual advergames on adolescents’ brand attitudes | 405 | Mixed, 11-17 years | Advergame | Brand attitude  Enjoyment of the game | · The comparison between the game with less conspicuous placement and the control game was significant (p < .03), suggesting that the subtle placement of M&M's provoked a significant improvement in the brand attitude.  - The comparison between the game with more conspicuous placement and the control game was significant (p < .01), suggesting that an effective improvement of the attitude to M&M's was caused by the prominent placement | Fair |
| Rifon et al. (2014), United States | Age-dependent effects of food advergame brand integration and interactivity | 276 | Mixed, 5-10 years | Advergame | Attitude towards the game    Brand recognition    Persuasion knowledge   Brand request   Brand attitude   Perceived healthiness of brand | - Brand integration significantly influenced recognition (yes or no to seeing a branded cereal box in the game (p < .001)  - The highest incidence of children reporting recognition was in the Integrated condition (79.8%), followed by the Background condition (68.8%)  - Recognition was dependent on age (p < .01) with a higher incidence of older children (83.3%) than younger children (66.3%) reporting having seen one of the cereal boxes  - Playing the game (81.9%) created a higher incidence of recognising one of the boxes (p < .02) than watching the game (65.9%), and the effect was not age dependent.  - There were no significant main effects on attitude towards the brand (p = 0.11).  - Persuasion knowledge levels were low, with 79.4% of all the children scoring 0/2 on the index  - Persuasion knowledge was age dependent, with older children displaying greater knowledge than younger children (p < .01). Brand Integration (p < .02) and Interactivity (Play/watch) (p < .02) had significant effects on the activation of persuasion knowledge.  - Playing the game generated greater persuasion knowledge than watching (p < .02) | Good |
| Vanwesenbeeck et al. (2014), Belgium | Children and advergames: the role of product involvement, prior brand attitude, persuasion knowledge and game attitude in purchase intentions and changing attitudes | 279 | Mixed, 10-12 years | Advergame | Brand attitude   Purchase intention   Persuasion knowledge   Game attitude | · Prior brand attitude is an important factor in the effectiveness of advergames aimed at children - children with a positive prior brand attitude showed a positive attitude change in this study ( p < .01)  - Children in the negative attitude change group contained a higher percentage of respondents who had a neutral prior brand attitude (p < .01)  - Game attitude was found to have been more favourable among children whose attitudes changed positively (p < .01) than among children whose attitudes changed negatively  - The degree of attitude change was positively inﬂuenced by the child’s attitude towards the game (p < .01)  - The degree of product involvement (low/high) did not inﬂuence the level of attitude change after game play | Good |
