# Supplementary material for: Food Marketing Influences Children’s Attitudes, Preferences and Consumption: A Systematic Critical Review
Source: Nutrients. 2019 Apr 18;11(4):875. doi: 10.3390/nu11040875 (PMC6520952; doi:10.3390/nu11040875)
Supplement: Supplementary file 1 [file nutrients-11-00875-s001.zip › Supplementary Files/Supplementary table S2-Endorsers.docx]

Endorsers

| **Author (year), country** | **Title** | **Sample size** | **Participant characteristics (sex, age)** | **Main marketing technique/vehicle used** | **Outcome measures** | **Primary outcomes/themes** | **Quality Assessment** |
| --- | --- | --- | --- | --- | --- | --- | --- |
| Boyland et al. (2013), United Kingdom | Food choice and overconsumption: Effect of a premium sports celebrity endorser | 181 | Mixed, 8-11 years | Celebrity endorsement | Energy intake | - Children who viewed the endorsed commercial, another food commercial or the TV footage of the endorser outside of a food context consumed, significantly more of the Walker's chips compared with children in other groups (p < .001) - Children did not reduce their intake of the supermarket brand product to compensate; thus, the endorser effect contributed to overconsumption. | Good |
| Dixon et al. (2014), Australia | Effects of nutrient content claims, sports celebrity endorsements and premium offers on pre-adolescent children's food preferences: experimental research | 1302 | Mixed, grade 5-6 | Celebrity endorsement | Food choice  Perceptions on nutritional content  Product ratings | - Compared to the control condition, children were more likely to choose unhealthy products featuring nutrient content claims (both genders) (p < .001) and sports celebrity endorsements (boys only) (p < .03)  - Perceptions of nutritional content were enhanced by nutrient content claims (p < .001) | Good |
| Putnam et al. (2018), United States | Character apps for children's snacks: effects of character awareness on snack selection and consumption patterns | 132 | Mixed, 4-5 years | Media characters (in apps) | Food choice   Awareness of character | - An ordered logistic regression found no significant effect of treatment conditions compared with the control group. - Within treatment conditions, awareness of the character led to selection and consumption of more healthy snacks in the healthier condition (p < .01), and of unhealthy snacks in the unhealthy condition (p < .03), but children were unaware that the character influenced their decisions. | Good |
| Smits et al. (2012), Belgium | Endorsing children’s appetite for healthy foods: Celebrity versus non-celebrity spokes-characters | 57 | Mixed, 6-7 years | Celebrity endorsements and promotional characters | Frequency of consumption  Frequency of purchase requests  Hunger | - Adding a spokes-character (i.e., a gnome) to a food product increases the appetite, the (intended) frequency of consumption and the (intended) frequency of parent requests for that product among 6- to 7-year-old children, for both unhealthy and healthy foods (p < .04) - The effect of the celebrity spokes-character is in all cases greater than the effect of a similar (but unknown) gnome. | Fair |
