# Supplementary material for: Food Marketing Influences Children’s Attitudes, Preferences and Consumption: A Systematic Critical Review
Source: Nutrients. 2019 Apr 18;11(4):875. doi: 10.3390/nu11040875 (PMC6520952; doi:10.3390/nu11040875)
Supplement: Supplementary file 1 [file nutrients-11-00875-s001.zip › Supplementary Files/Supplementary table S3-Internet.docx]

Internet

| **Author (year), country** | **Title** | **Sample size** | **Participant characteristics (sex, age)** | **Main marketing technique/vehicle used** | **Outcome measures** | **Primary outcomes** | **Quality Assessment** |
| --- | --- | --- | --- | --- | --- | --- | --- |
| Pettigrew et al. (2013), Australia | The effects of television and Internet food advertising on parents and children | 2604 | Mixed, 8-14 years and their parents | Television commercial and social media | BMI  Brand preference  Brand attitude   Frequency of consumption | · Product evaluations were more favourable among the advertising exposure groups relative to the control group (p < .001) | Good |
| Tarabashkina et al. (2016),  Australia | Food advertising, children’s food choices and obesity: interplay of cognitive defences and product evaluation: an experimental study | 354 | Mixed, 7-13 years | Internet pop-up advertisement | BMI   Food choice   Product evaluations   Persuasion knowledge    Nutritional knowledge | - Children in the experimental group chose biscuit 1 more frequently compared with children in the control group (not significant – p = 0.63) | Good |
