# Supplementary material for: Food Marketing Influences Children’s Attitudes, Preferences and Consumption: A Systematic Critical Review
Source: Nutrients. 2019 Apr 18;11(4):875. doi: 10.3390/nu11040875 (PMC6520952; doi:10.3390/nu11040875)
Supplement: Supplementary file 1 [file nutrients-11-00875-s001.zip › Supplementary Files/Supplementary table S4-Packaging.docx]

Packaging

| **Author (year), country** | **Title** | **Sample size** | **Participant characteristics (sex, age)** | **Main marketing technique/vehicle used** | **Outcome measures** | **Primary outcomes/themes** | **Quality Assessment** |
| --- | --- | --- | --- | --- | --- | --- | --- |
| Ares et al. (2016), Uruguay | Influence of label design on children’s perception of two snack foods: Comparison of rating and choice-based conjoint analysis | 238 | Mixed, grades 1-6 | Promotional characters and labelling | Evaluation of labels   Food preference | - Cartoon characters influenced preference of yoghurt (p < .01) and sponge cake (p < .001)  - Nutrition claims influenced preference of yoghurt (p < .001) and sponge cake (p < .001)  - Front of pack nutritional information did not significantly influence preference of foods | Fair |
| Arrua et al. (2017), Uruguay | Influence of label design on children's perception of 2 snack foods | 221 | Mixed, 9-13 years | Promotional characters | Product attitude | - Low-income children showed a more positive attitude toward the yoghurt and sponge cake than did middle- and high-income children (p < .001) -The inclusion of cartoon characters on food labels is associated with fun (p < .05)  - Low-income children seem more susceptible to the marketing strategies of food companies than do middle- and high-income children | Fair |
| Dixon et al. (2014), Australia | Effects of nutrient content claims, sports celebrity endorsements and premium offers on pre-adolescent children's food preferences: experimental research | 1302 | Mixed, grade 5-6 | Labelling | Food choice Perceptions on nutritional content Product ratings | - Compared to the control condition, children were more likely to choose unhealthy products featuring nutrient content claims (both genders) (p < .001) and sports celebrity endorsements (boys only) (p < .03)  - Perceptions of nutritional content were enhanced by nutrient content claims (p < .001) | Good |
| Elliott et al. (2013), Canada | Food branding and young children’s taste preferences: A reassessment | 65 | Mixed, 3-5 years | Branding | Taste preference | - Children preferred the taste of the McDonald's fries (p < .05) and McDonald's carrots (p < .05) to the plain-wrapped equivalent.  - For the McDonald's versus coloured wrapping "pair", children preferred the taste of the carrots in coloured wrapping (p < .04).  - For the remaining 12 food/packaging combinations, the results are not statistically significant.  - Results suggest that children rely more on aesthetics than on familiar branding when making their choices | Fair |
| Forman et al. (2009), United States | Food branding influences ad libitum intake differently in children depending on weight status. Results of a pilot study | 43 | Mixed, 4-6 years | Branding | BMI   Energy Intake   Brand awareness | - OW children consumed significantly more energy per meal (nearly 200 kcal more, regardless of condition) than non-OW (p < .001) - Child age and brand awareness were positively associated (p < .001)  - There were no differences in intake at the branded vs. unbranded conditions (p = 0.8) - OW children consumed an additional 40kcal in branded vs. unbranded meals whereas non-OW children consumed 45kcal less in branded meals (p < .05) | Good |
| Gregori et al. (2013),  Latin America | Investigating the obesogenic effects of marketing snacks with toys: an experimental study in Latin America | 660 | Mixed, 3-10 years | Toys | BMI    Energy intake | - The inclusion of toys in food packages was not shown per se to lead to an increase in the caloric intake of children (p = 0.28) | Good |
| Gregori et al. (2014), India | Food packaged with toys: an investigation on potential obesogenic effects in Indian children | 1,680 | Mixed, 3-11 years | Toys | BMI   Brand awareness   Energy intake | - Food consumption was not influenced by added toys, even after adjustment for potential confounding factors (p = 0.9) | Good |
| Josion-Portail (2012), France | Children, packaging and on-pack nutritional information: An exploratory study | 10 | Mixed, 7-12 years | Labelling | Product description  Product attitude  Willingness to try the product  Product evaluation | - Children between 7 and 12 years old perceive nutritional information available on product packages and refer to them when evaluating products in terms of healthiness. - Children’s perceptions and understanding of nutritional information are not always correct. - Nutritional elements do not seem to be a key driver in children’s demand for the product. | Good |
| Keller et al. (2012), United States | The impact of food branding on children's eating behaviour and obesity | Study 1: 43 participants    Study 2: 41 participants    Study 3: 16 | Study 1: Mixed, 4-6 years    Study 2: Mixed, 7-9 years    Study 3: Mixed, 4-5 years | Branding | Energy intake   BMI   Cognitive bias | Study 1  - OW children consumed about ~ 41 kcal more when foods were branded, while non-OW children consumed about ~ 45 kcal less when foods were branded (p < .05) compared to when they were unbranded.  - No differences between sex (p = 0.40)  Study 2  - The Food Brand Stroop Task demonstrated that OW children may have a cognitive bias toward some food brand images (p < .05)  - All children, regardless of weight status, ate more at the branded compared to the unbranded meal (p <.07).  - There were no significant differences in intake between conditions depending on whether the food was healthy or unhealthy  - Girls ate 100 kcal more when the meals were branded than when they were unbranded (p < .05)  Study 3  - Children in the intervention group consumed more servings of both fruits and vegetables across the three study time points: baseline, treatment, and control (p < 0.05 for all) | Good |
| Kellershohn et al. (2018), United Kingdom | Young children’s perceptions of branded healthy fast food | 20 | Mixed, 4-6 years | Branding | Food choice   Taste   Perceived healthiness   Perceived food choice of mother | - Children have a strong opinion of what food items will taste good, which items are healthy and which items a parent might want them to eat and these classifications, while they may overlap, are distinct to them - Brand logos influenced some of the children’s perceptions on how a common food item would taste. However, the branding did not elevate the perception of the apple slices in the bags, in terms of taste or healthiness compared to the whole fruit. | Good |
| Kotler et al. (2012), United States | The influence of media characters on children's food choices | 343 | Mixed, 2-6 years | Promotional characters | Food preference  Food choice  Energy intake | - When foods within the same category (i.e., two vegetables) were competing against each other, children were more likely to indicate a preference for the target food when a Sesame Street character was associated with that food compared with no character or an unknown character, the latter of which did not differ significantly from each other (p < .01) - When the target food was a sugary or salty snack, Sesame Street fans were more likely to choose that food when a Sesame Street character was associated with it compared with no character or an unknown character (the latter of which did not differ significantly from each other) (p < .01)  - Sesame Street fans were not statistically more likely to choose a healthy food over a sugary or salty snack even when the healthier food was associated with a Sesame Street character.  - Children were more likely to try the alternative food when a Sesame Street character was associated with that food than when an unknown character was associated with that food (p < 0.5) | Good |
| Lapierre et al. (2011), United States | Influence of licensed spokes characters and health cues on children's ratings of cereal taste | 80 | Mixed, 4-6 years | Promotional characters | Taste  Food preference | - Children who saw a popular media character on the box reported liking the cereal more than those who viewed a box without a character on it (p < .01)  - Children who sampled the cereal named Healthy Bits reported that they enjoyed the cereal more than children who were given the same cereal with the name Sugar Bits (p < .04)  - Children who received the cereal named Sugar Bits with no character on the box reported enjoying the cereal's taste significantly less than children in each of the other 3 groups (p < .04) | Fair |
| Letona et al. (2014), United States | Effects of licensed characters on children’s taste and snack preferences in Guatemala, a low/middle income country | 121 | Mixed, 4-11 years | Promotional characters | Food preference   Taste   Character identification | - Children were signiﬁcantly (p < .001) more likely to prefer the taste of the foods inside the package with the licensed character compared with the one with no character  - Most children (66%) chose the food in the package with the character for a snack.  - Younger children (p < .001) were more likely to prefer the taste of the food inside the package with the character. | Good |
| Marshall et al. (2006), United Kingdom | Examining the relationship between product package colour and product selection in pre-schoolers | 43 | Mixed, 3-5 years | Colour | Product choice  Favourite colour | - Favourite colours are related to package colour selection for preschool children. The results showed a high correlation between favourite colour and choice of product across the total sample (p < .001) | Fair |
| McGale et al. (2016), United Kingdom | The influence of brand equity characters on children's food preferences and choices | 209 | Mixed, 4-8 years | Promotional characters | Taste preference   Character identification/likeability   Food choice | - Children signiﬁcantly preferred both Cheestrings (p < .001) and CocoPops SnackBars (p < .03) when a brand equity character was displayed on the packaging, compared with the same food presented in a package without the character.  - This effect was not seen for Pom-Bear Potato Snacks (p > .05)  - Children were also signiﬁcantly more likely to choose a food item with an incongruent brand-equity character displayed on the packaging than those without a brand equity (p < .001) | Good |
| Miller at al. (2011), United States | Children’s use of on‐package nutritional claim information | 124 | Mixed, 8-12 years | Labelling | Food choice   Perceived healthfulness | - The presence of an on‐package claim in the choice set led to unhealthier choices (p < .01)    - General claims still led to avoidance and an increased propensity to choose less healthful products (p < .02) | Fair |
| Ogle et al. (2017), United States | Influence of cartoon media characters on children's attention to and preference for food and beverage products | 149 | Mixed, 6-9 years | Promotional characters | Visual attention    Product choice | - Children pay more attention to products with (vs without) characters (p < .001)  - Children prefer less- (vs more-) healthful products (p < .001)  - Children prefer products with (vs without) characters (p < .001)  - Children prefer more-healthful products with characters over less-healthful products without characters (p < .001) | Good |
| Roberto et al. (2010), United States | Influence of licensed characters on children’s taste and snack preferences | 40 | Mixed, 4-6 years | Promotional characters | Taste preference  Snack preference   Identification of cartoon character | - Children preferred the taste of a food when a licensed character appeared on it (p < .001)  - Children were signiﬁcantly more likely to choose licensed-character food items for snacks (p < .001) | Good |
| Robinson et al. (2007), United States | Effects of fast food branding on young children\'s taste preferences | 63 | Mixed, 3-5 years | Branding | Brand recognition   Taste Product preference | - Low-income preschool children preferred the tastes of foods and drinks if they thought they were from McDonald’s (p < .001) demonstrating that brand identity can influence young children’s taste perceptions. This was true even for carrots, a food that was not marketed by or available from McDonald’s | Good |
| Smits et al. (2012), Belgium | Endorsing children’s appetite for healthy foods: Celebrity versus non-celebrity spokes-characters | 57 | Mixed, 6-7 years | Celebrity endorsements and promotional characters | Frequency of consumption  Frequency of purchase requests  Hunger | - Adding a spokes-character (i.e., a gnome) to a food product increases the appetite, the (intended) frequency of consumption and the (intended) frequency of parent requests for that product among 6- to 7-year-old children, for both unhealthy and healthy foods (p < .04) - The effect of the celebrity spokes-character is in all cases greater than the effect of a similar (but unknown) gnome. | Fair |
| Ülger (2009), Turkey | Packages with cartoon trade characters versus advertising: An empirical examination of pre-schoolers’ food preferences | 144 | Mixed, 6 years | Promotional characters | Product choice | · Both the participants who watched the CD with Product B commercials and the ones who watched it without commercials preferred Product A - the one with the child-appeal package (73.6% vs. 26.3%). | Fair |
