# Supplementary material for: Food Marketing Influences Children’s Attitudes, Preferences and Consumption: A Systematic Critical Review
Source: Nutrients. 2019 Apr 18;11(4):875. doi: 10.3390/nu11040875 (PMC6520952; doi:10.3390/nu11040875)
Supplement: Supplementary file 1 [file nutrients-11-00875-s001.zip › Supplementary Files/Supplementary table S5-Print Advertising.docx]

Print Advertising

| **Author (year), country** | **Title** | **Sample size** | **Participant characteristics (sex, age)** | **Main marketing technique/vehicle used** | **Outcome measures** | **Primary outcomes/themes** | **Quality Assessment** |
| --- | --- | --- | --- | --- | --- | --- | --- |
| Jones et al. (2011), Australia | An experimental study on the effects of exposure to magazine advertising on children’s food choices | 47 | Mixed, 5-12 years | Magazine | Food choice  Attitudes towards advertising format | · Children in the experimental group chose more advertised food items (p < .05) and less of the non-advertised unhealthy food items (p < .04).  - Girls were more likely to state that it was important that a snack food be healthy (p < .01), and boys that it taste good (p < .03).  - The participants showed generally positive attitudes towards magazine advertising  - Younger participants were more likely to state that they like magazine advertisements (p < .001) and that they think magazine advertisements are great (p < .001). | Fair |
| Jones et al. (2010), Australia | Like me, want me, buy me, eat me’: relationship-building marketing communications in children’s magazines | 10 | Mixed, 6-13 years | Magazine | Brand recognition   Attitude to advertising format  Brand attitude | · Children notice, like and are receptive to the marketing strategies used in these magazines  - The participants were attracted to and liked this union of well-known characters with popular products.  - The participants made clear associations between brands and social or physical outcomes  - Children did not identify the advertisement pages as ‘advertisements’ but rather as ‘information’  - When shown samples of children’s magazines and asked what parts of the magazines they liked, the participants nominated the quizzes, puzzles and/or games (n 3); movie references (n 3); posters (n 2); stories (n 2); references to famous people (n 2); cute stuff (n 1); bright colours (n 1); advertisements for toys (n 1); comics (n 1); references to computer games (n 1); and articles (n 1)  · While younger children have less of an understanding of the intent of most magazine advertisements, older children appear to be susceptible to more sophisticated marketing strategies. | Good |
| King et al. (2008), United Kingdom | Magazine adverts for healthy and less healthy foods: Effects on recall but not hunger or food choice by pre-adolescent children | 309 | Mixed, 9-10 years | Magazine | Self-perception  Mood  Hunger  Body shape satisfaction   Food choice   BMI | · There was no effect of advert group on food choice (p = 0.35)  - No group differences in self-perception, mood or hunger were found after viewing printed adverts for healthy or less healthy foods, or compared with non-food adverts    · Recall was significantly higher for less healthy foods, even when estimated prior exposure to advertisements was accounted for (p < .001) | Good |
