# Supplementary material for: Food Marketing Influences Children’s Attitudes, Preferences and Consumption: A Systematic Critical Review
Source: Nutrients. 2019 Apr 18;11(4):875. doi: 10.3390/nu11040875 (PMC6520952; doi:10.3390/nu11040875)
Supplement: Supplementary file 1 [file nutrients-11-00875-s001.zip › Supplementary Files/Supplementary table S6-Television.docx]

Television/movies

| **Author (year), country** | **Title** | **Sample size** | **Participant characteristics (sex, age)** | **Main marketing technique/vehicle used** | **Outcome measures** | **Primary outcomes/themes** | **Quality Assessment** |
| --- | --- | --- | --- | --- | --- | --- | --- |
| Anschutz (2009), The Netherlands | Side effects of television food commercials on concurrent non-advertised sweet snack food intakes in young children | 120 | Mixed, 8-12 years | Television commercials | Energy intake  Liking of test food | - No main effect of commercial condition on food intake (p = 0.38) - Interaction commercial condition × sex on food intake (p < .05)  - No difference in liking of test food across condition (p = 0.60) | Good |
| Anderson et al. (2015), Canada | Mealtime exposure to food advertisements while watching television increases food intake in overweight and obese girls but has a paradoxical effect in boys | 23 | Mixed, 9-14 years | Television commercials | Energy intake | - Food ads increased energy intake at the meal in OW/OB girls (p < .03)  - Both boys and girls reduced energy intake at the meal in compensation for energy in the glucose beverage (p < 0.05) | Good |
| Anschutz et al. (2010), The Netherlands | Maternal encouragement to be thin moderates the effect of commercials on children's snack food intake | 121 | Mixed, 8-12 years | Television commercials | Energy intake   Maternal encouragement to be thin | - Children who perceived maternal encouragement to be thin ate slightly more when exposed to energy-dense food commercials and especially when exposed to light food commercials than when exposed to neutral commercials (p < .001)  - No main effect of commercial condition on food intake (p = 0.20) | Good |
| Auty et al. (2004), United Kingdom | Exploring children's choice: The reminder effect of product placement | 105 | Mixed, 6-12 years | Product placement in movies | Drink choice | -Those who had seen the branded clip made a significantly different choice of drink (experimental group more likely to choose Pepsi or Coke than control group) (p < .04) - The responses to the interviews suggest that it is not simply exposure to the film but rather previous exposure together with a reminder in the form of recent exposure that affects choice. - Age (and by implication processing skill) does not appear to be a mediating factor affecting choice, because implicit memory (mere exposure) seems to be more important than explicit recall | Fair |
| Borzekowski et al. (2001), United States | The 30-second effect: an experiment revealing the impact of television commercials on food preferences of pre-schoolers | 46 | Mixed, 2-6 years | Television commercials | Food choice | - Children who were exposed to a videotape with embedded commercials were significantly more likely to choose the advertised items than children who saw the same videotape without commercials (p < .01) | Fair |
| Boyland et al. (2008), United Kingdom | Beyond-brand effect of television food advertisements on food choice in children: the effects of weight status | 59 | Mixed, 9-11 years | Television commercials | BMI   Energy intake  Food choice | - Exposure to food adverts produced substantial and significant increases in energy intake in all participants (p < .001) - The increase in intake was largest in obese children (471 kcal) (p < .04) - All children increased their consumption of high-fat and/or sweet energy-dense snacks in response to the adverts (p < ·001)  - There was a signiﬁcant interaction between food choice and advert condition whereby food advert exposure increased the intake of all food items (except Snack-a-Jacks) in the group as a whole (p < .001) | Good |
| Boyland et al. (2015), United Kingdom | Exposure to 'healthy' fast food meal bundles in television advertisements promotes liking for fast food but not healthier choices in children | 59 | Mixed, 7-10 years | Television commercials | BMI  Nutritional knowledge   Liking of food advertised | - Children's liking for fast food, in general, increased after exposure to the food adverts relative to control (p < .01)  - Compared to children with high nutritional knowledge, those with low scores selected meals of greater energy content (305 kJ) after viewing the food adverts (p < .016)  - Exposure to adverts for 'healthy' meal bundles did not drive healthier choices in children, but did promote liking for fast food. | Good |
| Brown et al. (2017), United States | Influence of product placement in children's movies on children's snack choices. | 114 | Mixed, 9-11 years | Product placement | Food intake   Food choice   BMI   Hunger rating | - Participants consumed an average of 800.8 kcal; mean kcal eaten did not vary by movie watched (p = 0.8) - Participants who watched the high product-placement movie had 3.1 times the odds (95% CI 1.3-7.2) of choosing cheese balls (most featured snack) compared to participants who watched the low product-placement movie (p < .01) - Children's weight status did not significantly affect their choice of snack. | Good |
| Bruce et al. (2016), United States | The influence of televised food commercials on children's food choices: Evidence from ventromedial prefrontal cortex activations | 23 | Mixed, 8-14 years | Television commercials | BMI   Ratings of food   Ratings of commercials | - Watching food commercials changes the way children consider the importance of taste when making food choices. - Children did not use health values for their food choices, indicating children's decisions were largely driven by hedonic, immediate rewards (i.e., "tastiness"); however, children placed significantly more importance on taste after watching food commercials compared with non-food commercials (p < .05). This change was accompanied by faster decision times during food commercial trials (p < .05) - The ventromedial prefrontal cortex, a reward valuation brain region, showed increased activity during food choices after watching food commercials compared with after watching non-food commercials (p < .05) | Fair |
| Chernin (2008), United States | The effects of food marketing on children's preferences: Testing the moderating roles of age and gender | 133 | Mixed, 5-11 years | Television commercials | Product preference | - Exposure to food commercials increased children’s preferences for the advertised products (p < .01) - Age did not moderate this effect; younger and older children were equally persuaded by the commercials (p = 0.36) - Boys were more influenced by the commercials than girls (p < .03) | Good |
| Dixon et al. (2017), Australia | Food marketing with movie character toys: Effects on young children's preferences for unhealthy and healthier fast food meals | 904 | Mixed, 5-9 years | Product placement/movie tie-ins | Food choice   Product perception | - Children showed a preference for unhealthy meals over healthier ones (76%)  - Children were more likely to select a given meal type when it was paired with a MTIP (movie tie-in premium).  - Children were significantly more likely to select a healthier meal over an unhealthy meal when only the healthier meals were accompanied by a MTIP (p < .001)  - Children were significantly more likely to select a unhealthy meal paired with a MTIP over a unhealthy meal without a MTIP (p < .001)  - When healthier meals were accompanied by a MTIP, children reported the meal looked better (p < .04), would taste better (p < .02), they would be more likely to ask their parents for this meal (p < .03), and they would feel happier if their parents bought them this meal (p < .01), compared to when the healthier meal was not accompanied by a MTIP | Good |
| Dixon et al. (2007), Australia | The effects of television advertisements for junk food versus nutritious food on children's food attitudes and preferences | 919 | Mixed, grade 5-6 | Television commercials | Food attitude  Food preference | - There was no support for the hypothesis that children exposed to junk food ads would show enhanced attitudes and intentions favouring unhealthy foods compared to children not exposed to junk food ads. | Good |
| Emond et al. (2016), United States | Randomized exposure to food advertisements and eating in the absence of hunger among pre-schoolers | 60 | Mixed, 2-5 years | Television commercials | BMI   Hunger   Liking of snack    Energy intake | - Child BMI was not related to eating in the absence of hunger (EAH). - Mean kilocalories consumed during the EAH phase was greater among children exposed to the food advertisements versus those exposed to the non-food advertisements) (p < .04), an effect driven by greater consumption of the advertised food (p < .01) | Good |
| Esmaelipour et al. (2018), Iran | Children’s food choice: Advertised food type, health knowledge and entertainment | 330 | Mixed, 6-11 years | Television commercials & advergames | Food choice | - Children chose more unhealthy foods after exposure to unhealthy food advertising (p < .001) - This effect was greater for a higher level of entertainment (p < .01), and was successfully moderated by the activation of health knowledge (p < .03) | Fair |
| Ferguson (2014), United States | Advertising and fictional media effects on healthy eating choices in early and later childhood | 304 | Mixed, 3-12 years | Television commercials | BMI   Food choice | - Young children (3-5) were influenced by media clips of food whether advertisements or fictional (p < .05) - Middle elementary children (6–8) were influenced mainly by their parents (p < .01)  - Older children (9+) were influenced by their parents but marginally so (p = .055) and were not influenced by media exposure or parental influence. | Fair |
| Ferguson et al. (2012), United States | Advertising influences on young children's food choices and parental influence | 75 | Mixed, 3-8 years | Television commercials | BMI   Food choice | - Children were more likely to choose the advertised item despite parental input (p < .001) | Fair |
| Gatou et al. (2016), Greece | The short‐term effects of television advertisements of cariogenic foods on children's dietary choices | 183 | Mixed, 11-12 years | Television commercials | Advertisement recall    Advertisement recognition    Food choice    DMFT (decayed missing and filled teeth)   Dietary habits/leisure activities | - Exposure to food advertisements significantly decreased the selection of healthy foods (p < .05)   - Children who spent more time watching television chose an increased number of healthy foods, after their exposure to food advertisements (p < .03) - Regardless of condition, girls selected fewer foods than boys (p < .001) - Children with a higher DMFT index selected more unhealthy foods than children with a lower DMFT index (p < .02) - Obese children chose an increased number of healthy foods than those who were overweight (p < .04) and normal weight (p < .001) | Good |
| Gilbert-Diamond (2017), United Kingdom | Television food advertisement exposure and FTO rs9939609 genotype in relation to excess consumption in children | 172 | Mixed, 9-10 years | Television commercials | Energy intake    Hunger rating  Fat mass and associated gene   BMI | - Participants who viewed food advertisements consumed an average of 48 kcals more of a recently advertised food than those who viewed toy advertisements (p < .01) - There was a statistically significant interaction between genotype and food advertisement condition (p < .02) where the difference in consumption of a recently advertised food related to food advertisement exposure increased linearly with each additional FTO risk allele, even after controlling for body mass index percentile | Good |
| Goldberg et al. (1978), United States | Tv messages for snack and breakfast foods: Do they influence children's preferences? | 80 | Mixed, first grade | Television commercials | Food choice   Perception of healthy and unhealthy foods | - The participants who viewed the sugary commercials chose more sugary foods than those who viewed the PSA’s and no commercials at all (p < .05) - There was no significant difference between levels of exposure (4.5 vs 9 minutes). | Poor |
| Gorn et al. (1980), Canada | Children's responses to repetitive television commercials | 151 | Boys, 8-10 years | Television commercials | BMI  Food choice   Energy intake | - Brand preference for advertised brand influenced by commercial (p < .05)  - Exposure to commercials was not effective in increasing consumption of ice-cream ( p = 0.10) | Poor |
| Gorn et al. (1982), Canada | Behavioural evidence of the effects of televised food messages on children | 288 | Mixed, 5-8 years | Television commercials | Food choice   Energy intake   Attitudes to foods | - Children who viewed candy commercials chose significantly more candy over fruit as snacks (p < .001) - Children who viewed candy commercials (which included Kool Aid) chose significantly less orange juice than Kool Aid (p < .05) | Poor |
| Halford et al. (2004), United Kingdom | Effect of television advertisements for foods on food consumption in children | 42 | Mixed, 9-11 years | Television commercials | TV commercials and recognition   Food choice   Energy intake    Assessment of externality    BMI | - Obese and overweight children recognised significantly more food ads than the lean children (p < .001)  - The obese and overweight groups ate significantly more than the healthy-weight group (p < .001)  - The ability to recognise the food commercials significantly correlated with the amount of food eaten after exposure (p < .001) - There was no significant variation among the lean, overweight or obese groups in the DEBQ external eating scores. | Good |
| Halford et al. (2007), United Kingdom | Beyond-brand effect of television (TV) food advertisements/commercials on caloric intake and food choice of 5-7-year-old children | 93 | Mixed, 5-7 years | Television commercials | Product recognition  BMI   Food choice   Energy intake | - Food advert exposure produced a significant increase in total food intake in young children (p < .001) - Exposure to food advertisement increases food intake in all children, but recognition of food commercials is related to body mass index (BMI) (p < 0.01) | Good |
| Halford et al. (2008), United Kingdom | Children's food preferences: effects of weight status, food type, branding and television food advertisements (commercials) | 37 | Mixed, 11-13 years | Television commercials | BMI   Advertisement recall list   Food preference   Food choice | - Normal weight children selected more branded and non-branded food items after exposure to food advertisements than in the control (toy advertisement) condition (p < .04) - Obese and overweight children showed a greater preference for branded foods than normal weight children per se (p < .03)  - For obese and overweight children there was a significant correlation between food advertisement recall and the total number of food items chosen in the experimental (food advertisement) condition (p < .03) | Good |
| Harris et al. (2018), United States | Food and beverage TV advertising to young children: Measuring exposure and potential impact | 84 | Mixed, 4-7 years | Television commercials | Brand attitude  Advertisement attitude | - Both pre-schoolers (4–5 years) and slightly older children (6–7 years) liked the child-directed food ads they viewed in this study (p < .01)  - There was a significant main effect of media experience on ad liking (p < .001)  - Despite companies' claims that these ads are directed to children ages 6 and older, there was no significant main effect of age on ad liking (p = 0.58), although previous product consumption may have affected brand liking for older children more than for younger children. Among younger children, 71% who had previously consumed the advertised product reported liking the brand, compared to 55% who had not previously consumed the product. This difference was greater for older children; 89% of older children who had previously consumed the advertised product reported liking the brand, while just 33% who had not consumed the product reported liking the brand. | Fair |
| Harris et al. (2018), United States | Teaching children about good health? Halo effects in child-directed advertisements for unhealthy food | 138 | Mixed, 7-11 years | Television commercials | Attitudes   Food choice   Energy intake | - Children in the health halo condition rated the advertised nutrient-poor products as significantly healthier compared with children in other conditions (p < .01), but the other commercials did not affect children's attitudes about other advertised products (p's > 0.5).  - Child age, gender or TV viewing habits did not significantly predict their ratings (p's > 0.18).  - There was no evidence that healthy lifestyle messages and/or healthy food commercials improved children's attitudes about nutrition, exercise or healthy snack consumption. | Good |
| Harris et al. (2009), United States | Priming effects of television food advertising on eating behavior | 118 | Mixed, 7-11 years | Television commercials | Energy intake   BMI   Children’s media habits   Liking of test food | - Children consumed significantly more food (45% more) (in general) when exposed to food advertising (p < .01) | Fair |
| Lorenzoni et al. (2017), Georgia | Effect of TV advertising on energy intake of Georgian children: Results of an experimental study | 60 | Mixed, 3-11 years | Television commercials | Energy intake   Brand awareness | · Findings from the present experimental study showed no association of TV viewing and TV advertising with energy intake in a sample of Georgian children (p = 0.29) | Good |
| Matthes et al. (2015), Austria | Children’s consumption behaviour in response to food product placements  in movies | 121 | Mixed, 6-14 years | Product placement | Food choice   Free brand recall   Brand attitude   Food intake | - Exposure to high-frequency product placements exerted a significant effect on snack consumption (p < .05), but no effect on brand or product attitudes. These effects were independent of children’s ages. | Good |
| Neyens et al. (2017), United Kingdom | Transferring game attitudes to the brand: persuasion from age 6 to 14 | 940 | Mixed, 6-14 years | Television commercial | Brand attitude  Intention to pester  Brand preference   Brand recognition  Source recognition   Source intent   Attitude towards advertising format | - Children who played the advergame reported significantly more positive **brand attitudes** compared to children who had watched the TV ad (p <.001), and children in the no advertising exposure control group (p < .03).  - For pester intent, the impact of the advertising format was also significant (p < .05).  - Pester intent was significantly higher for the advergame than the TV ad (p < .02), but not compared to the control group (p = 0.36. | Fair |
| Norman et al. (2018), Australia | Sustained impact of energy-dense TV and online food advertising on children’s dietary intake: a within-subject, randomised, crossover, counter-balanced trial | 160 | Mixed, 7-12 years | Television commercials and advergames | Brand recognition  BMI  Energy intake | - All children in the multiple-media condition ate more at a snack after exposure to food advertising compared with non-food advertising; this was not compensated for at lunch, leading to additional daily food intake of 194 kJ (p < .001).  - Exposure to multiple-media food advertising compared with a single-media source increased the effect on snack intake by a difference of 182 kJ (p < .01)  - Food advertising had an increased effect among children with heavier weight status in both media groups. | Good |
| Panic et al. (2013), Belgium | Comparing TV ads and advergames targeting children: the impact of persuasion knowledge on behavioural responses | 254 | Mixed, 7-10 years | Television commercials | Attitude toward the advertisement    Purchase requests  Understanding of the commercial source   Understanding persuasive intent | · **Children** liked the **advergame** (with cue) significantly more than the **TV** advertisement (p < .001)  - In the **TV** advertisement condition, the results show a significant negative effect of **persuasion knowledge** on purchase request (p <.03). | Fair |
| Pettigrew et al. (2013), Australia | The effects of television and Internet food advertising on parents and children | 2604 | Mixed, 8-14 years and their parents | Television commercial and internet advertising | Brand preference  Brand attitude   Frequency of consumption  BMI | · Product evaluations were more favourable among the advertising exposure groups relative to the control group (p < .001) | Good |
| Resnik et al. (1977), United States | Children’s television advertising and brand choice: A laboratory study | 45 parents responded | Mixed, 6-8 years | Television commercials | Food choice | - Children's brand choices were found to be influenced significantly by the communication of the commercial message for the previously-unknown brand (p < .02) | Fair |
| Toomey et al. (2013), United States | Branded product placement and pre-teenaged consumers: influence on brand preference and choice | 69 | Mixed, 8-12 years | Product placement | Brand preference  Food choice | · The placement of branded products in a television program developed for pre-teenagers may not influence attitudes or behaviour of the pre-teenaged consumers   · Pre-teens exposed to a branded product placement were not more likely to choose that brand immediately following exposure (p = 0.7) or two weeks later (p = 0.3). | Good |
| Ülger (2009), Turkey | Packages with cartoon trade characters versus advertising: An empirical examination of pre-schoolers’ food preferences | 144 | Mixed, 6 years | Television commercials | Product choice | · Both the participants who watched the CD with Product B commercials and the ones who watched it without commercials preferred Product A - the one with the child-appeal package (73.6% vs. 26.3%). | Fair |
| Uribe et al. (2015), Chile | The effects of TV unhealthy food brand placement on children. Its separate and joint effect with advertising | 483 | Mixed, 9, 12 & 15 years | Television commercials and product placement | Cognitive response    Affective response    Brand preference | · It was observed that the use of placement alone produced a significant increase on brand awareness when compared with the control group (p = .03)    · ‘Top of mind’ awareness of the brand increased from 57% to 63.8% when product placement was used with advertising (p < .04)  · The joint use of advertising and placement increased the disposition toward fast food from about 47% to 54% (p < .001). | Fair |
